# Effect of perioperative probiotic supplements on the short-term clinical outcomes of patients undergoing laparoscopic or robotic radical gastrectomy after neoadjuvant chemotherapy: Study protocol for a multicenter randomized controlled trial (GISSG2023 - 01 Study)

**DOI:** 10.1186/s12885-025-14115-x

**Published:** 2025-04-25

**Authors:** Gan Liu, Shougen Cao, Xiaodong Liu, Yulong Tian, Wenbin Yu, Jie Chai, Leping Li, Xixun Wang, Xianqun Chu, Quanhong Duan, Jianjun Qu, Hao Wang, Huanhu Zhang, Xinjian Wang, Xizeng Hui, Daogui Yang, Shaofei Zhou, Yinlu Ding, Hongbo Wang, Fengqiang Zhou, Baoguang Hu, Peiming Guo, Lixin Jiang, Guangyong Zhang, Qiang Pan, Xiaobin Zhou, Yanbing Zhou

**Affiliations:** 1 Department of Gastrointestinal Surgery, the Affiliated Hospital of Qingdao University, No. 16 Jiangsu Road, Qingdao, China; 2 Qilu Hospital of Shandong University, Jinan, China; 3 Shandong Cancer Hospital, Jinan, China; 4 Shandong Provincial Hospital, Jinan, China; 5 Yantai Yuhuangding Hospital, Yantai, China; 6 Shandong Jining No.1 People’s Hospital, Jining, China; 7 Affiliated Hospital of Weifang Medical University, Weifang, China; 8 Weifang People’s Hospital, Weifang, China; 9 Dongying People’s Hospital, Dongying, China; 10 Weihai Municipal Hospital, Weihai, China; 11 Weihai Central Hospital, Weihai, China; 12 Rizhao People’s Hospital, Rizhao, China; 13 Liaocheng People’s Hospital, Liaocheng, China; 14 Qingdao Municipal Hospital, Qingdao, China; 15 The Second Hospital of Shandong University, Jinan, China; 16 The People’s Hospital of Jimo, Qingdao, China; 17 Binzhou People’s Hospital, Binzhou, China; 18 Binzhou Medical University Hospital, Yantai, China; 19 Jinan Central Hospital, Jinan, China; 20 Yantai Yeda Hospital, Yantai, China; 21 Shandong Provincial Qianfoshan Hospital, Jinan, China; 22 Rushan People’s Hospital, Weihai, China; 23 Department of Epidemiology and Health Statistics, School of Public Health of Qingdao University, Qingdao, China

**Keywords:** Probiotics, Neoadjuvant chemotherapy, Gastric cancer, Postoperative infection, RCT

## Abstract

**Background:**

Gastric cancer is a common malignant tumor, and radical gastrectomy can markedly improve the prognosis of gastric cancer patients. However, some patients are diagnosed with advanced gastric cancer before receiving any antitumor therapy and need to receive neoadjuvant chemotherapy (NACT). Previous studies have shown that NACT may cause gut barrier dysfunction and intestinal dysbacteriosis which may further lead to infections. Probiotics have the potential to reduce postoperative infections and improve short-term outcomes after abdominal surgery; however, no large-sample, multicenter, randomized clinical trials have been conducted to explore the effectiveness of probiotics in gastric cancer patients receiving NACT. So we proposed a hypothesis that probiotics can improve short-term outcomes after minimally invasive radical gastrectomy in gastric cancer patients receiving NACT and designed this multicenter randomized controlled trial with the objective to verify this hypothesis.

**Methods/design:**

The GISSG 2023–01 study will be a prospective, open-label, multicenter RCT to verify whether perioperatively probiotic supplementation (begin from the end of the last cycle of NACT to postoperative day 7 or the discharge day) can reduce postoperative infections and improve recovery of gastrointestinal function and other short-term outcomes after minimally invasive radical gastrectomy in gastric cancer patients receiving NACT. A total of 318 patients who meet the inclusion criteria will be enrolled in this study and randomly divided into two groups in a 1:1 ratio: the probiotic group (*n* = 159) and the control group (*n* = 159). The participants in the probiotic group will receive perioperative probiotic supplementation, and those in the control group will receive blank control management. The other perioperative management protocols will be the same between the two groups. The primary outcome is postoperative infection compared between the two groups, and the secondary outcomes are postoperative recovery of gastrointestinal function, quality of life, laboratory parameters of systemic inflammation and other short-term outcomes.

**Discussion:**

The results of this RCT should clarify whether perioperative probiotic supplementation would reduce postoperative infection, promote recovery of gastrointestinal function, reduce laboratory parameters of systemic inflammation and improve symptoms and quality of life after minimally invasive radical gastrectomy in gastric cancer patients receiving NACT. It is hoped that our data will provide evidence that probiotic supplementation improves short-term outcomes in gastric cancer patients receiving NACT.

**Trial registration:**

This trial has been registered on https://clinicaltrials.gov/(NCT05901779).

**Supplementary Information:**

The online version contains supplementary material available at 10.1186/s12885-025-14115-x.

## Introduction

### Background and rationale

Gastric cancer is a common malignant tumor accounting for 5.6% of new cases and causes 7.7% new deaths of all global cases of cancer [1]. Some patients are diagnosed with advanced gastric cancer (AGC) before receiving any anticancer therapy, which poses challenges to the treatment and prognosis of these individuals. Although D2 radical gastrectomy is a standard and preferred surgical protocol for AGC [2], the high tumor burden, micrometastasis and high recurrence risk still impair the therapeutic effect of surgery.

At present, the treatment of locally AGC is centered on surgery and guided by multidisciplinary team advice [3]. Since the first report of applying NACT in treating gastric cancer [4], many studies have focused on the advantages of NACT. The MAGIC study showed that NACT can markedly shrink tumor size and prolong the overall survival, five-year survival rate and progression-free survival of patients with resectable adenocarcinoma of the stomach, esophagogastric junction, or lower esophagus [5]. The RESOLVE study provided evidence that compared with adjuvant CapOX (capecitabine combined with oxaliplatin), perioperative SOX (S- 1 combined with oxaliplatin) can improve the 3-year disease-free survival of patients with locally advanced gastric or gastroesophageal junction adenocarcinoma undergoing D2 gastrectomy [6]. Schuhmacher et al. demonstrated that NACT can shrink the tumor size, decrease the number of lymph node metastases, improve pN0 rate and improve the chance of R0 resection [7]. Although there are many advantages of NACT in treating AGC, it may increase postoperative complications such as incision infection and anastomotic leakage [7]. Our previous study showed that NACT may increase postoperative infections, leading to damage to gut barrier function (low expression of tight junction-related proteins and tight junction disruption) and gut dysbiosis (depletion of beneficial commensal bacteria and loss of microbiota diversity) [8]. 5-FU is widely used in NACT for gastric cancer. Previous studies showed 5-FU when given to rats has multiple effects on the gut and causes mucosal damage and disrupts the gut microbiota [9–11].

Probiotics are defined as “live microorganisms which when administered in adequate amounts confer a health benefit on the host” [12]. The specific mechanism of probiotic action is unclear, and current assumptions include direct antimicrobial activity and indirect competitive inhibition. Due to the side effects of NACT on the gut barrier and microbiota, many studies have focused on the functions of probiotics to explore their capacity to reverse those side effects. Yuan et al. found that *Bifidobacterium infantis* can maintain the normal mucosal structure, increase the expression of PCNA (proliferating cell nuclear antigen), and reduce the expression of NF-κB, proinflammatory factors and MPO (myeloperoxidase). These data suggest that the probiotic *B. infantis* is effective in reducing chemotherapy-induced intestinal mucositis in rats [9]. Justino et al. drew a similar conclusion as Yuan, although the probiotic they used was *Lactobacillus acidophilus* [10]*.* In surgical patients, intraoperative stress may cause an increase in intestinal permeability, gut dysbiosis and bacterial translocation, which are important pathogenic factors that increase postoperative infections. For this reason, the introduction of probiotics is expected to maintain the intestinal barrier function by decreasing abnormal intestinal permeability, reducing both intestinal inflammation and the release of cytokines and maintaining the dynamic balance of normal gut microbiota. The short-term effects of probiotics, for example the reduction of postoperative infections have been explored in pancreatobiliary surgery, liver transplant surgery, and colon and rectal surgery [13–20]. Although our previous single-center RCT appeared to suggest that perioperative probiotic supplements can reduce postoperative infections and improve recovery of gastrointestinal function in gastric cancer patients receiving neoadjuvant chemotherapy [21], these results need to be verified by a rigorously designed, large-sample RCT. There have been a large number of clinical trials exploring the effects of probiotics or synbiotics on the clinical outcomes of patients undergoing abdominal surgeries, but no multicenter, large sample RCT has focused on the effects of probiotics on the postoperative short-term clinical outcomes of gastric cancer patients receiving NACT.

### Objective

The aim of this study was to investigate the effects of probiotics on infectious complications, recovery of gastrointestinal function, laboratory parameters of systemic inflammation, quality of life and other short-term outcomes after laparoscopic or DaVinci robotic minimally invasive radical gastrectomy in gastric cancer patients who have completed NACT.

### Trial design

The GISSG 2023–01 study is designed as a multicenter open-label parallel-randomized superiority controlled clinical trial. All eligible participants will be randomized into a probiotic group and a blank control group in a 1:1 ratio. The intervention duration will begin from the end of the last cycle of NACT to postoperative day (POD) 7 or the discharge day.

## Methods

**Fig. 1:**
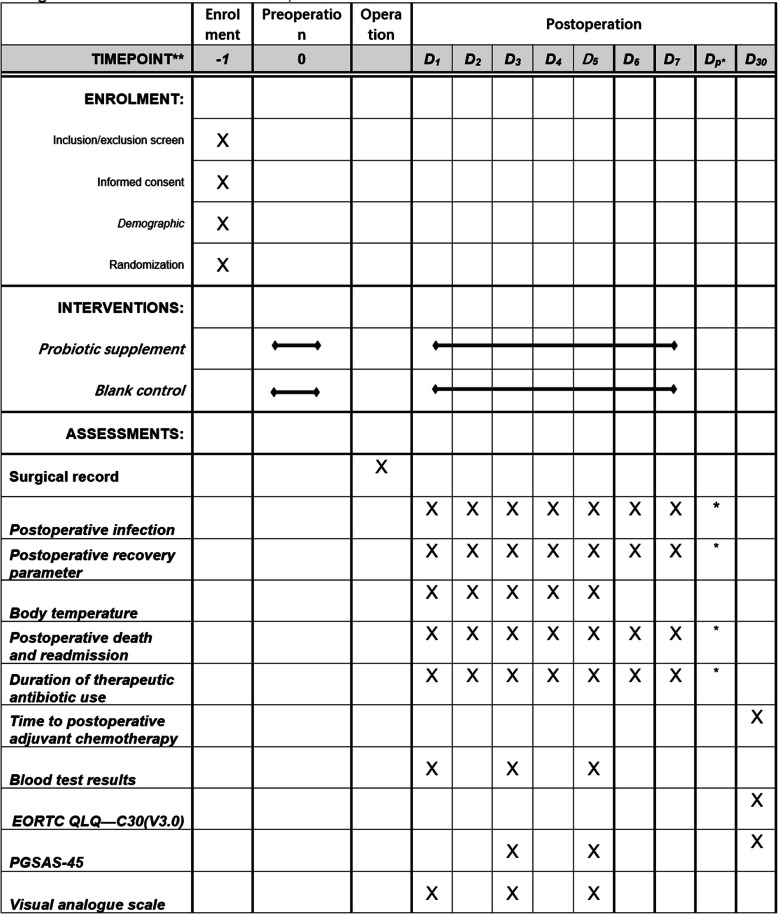
The enrollment, intervention and assessment items in the flowchart. The symbol × represents that the program needs to be collected. − 1, the end of the last cycle of preoperative chemotherapy; 0, from the end of the last cycle of preoperative chemotherapy to admission for surgery; D1, postoperative day 1; D2, postoperative day 2; D3, postoperative day 3; D4, postoperative day 4; D5, postoperative day 5; D6, postoperative day 6; D7, postoperative day 7; D30, postoperative day 30; Dp, prolonged postoperative hospital stay; * the majority of participants can be discharged before D7 under the ERAS perioperative management protocol, but if any situation prolongs the hospital stay, the marked items should be recorded and described on the CRF

**Fig. 2:**
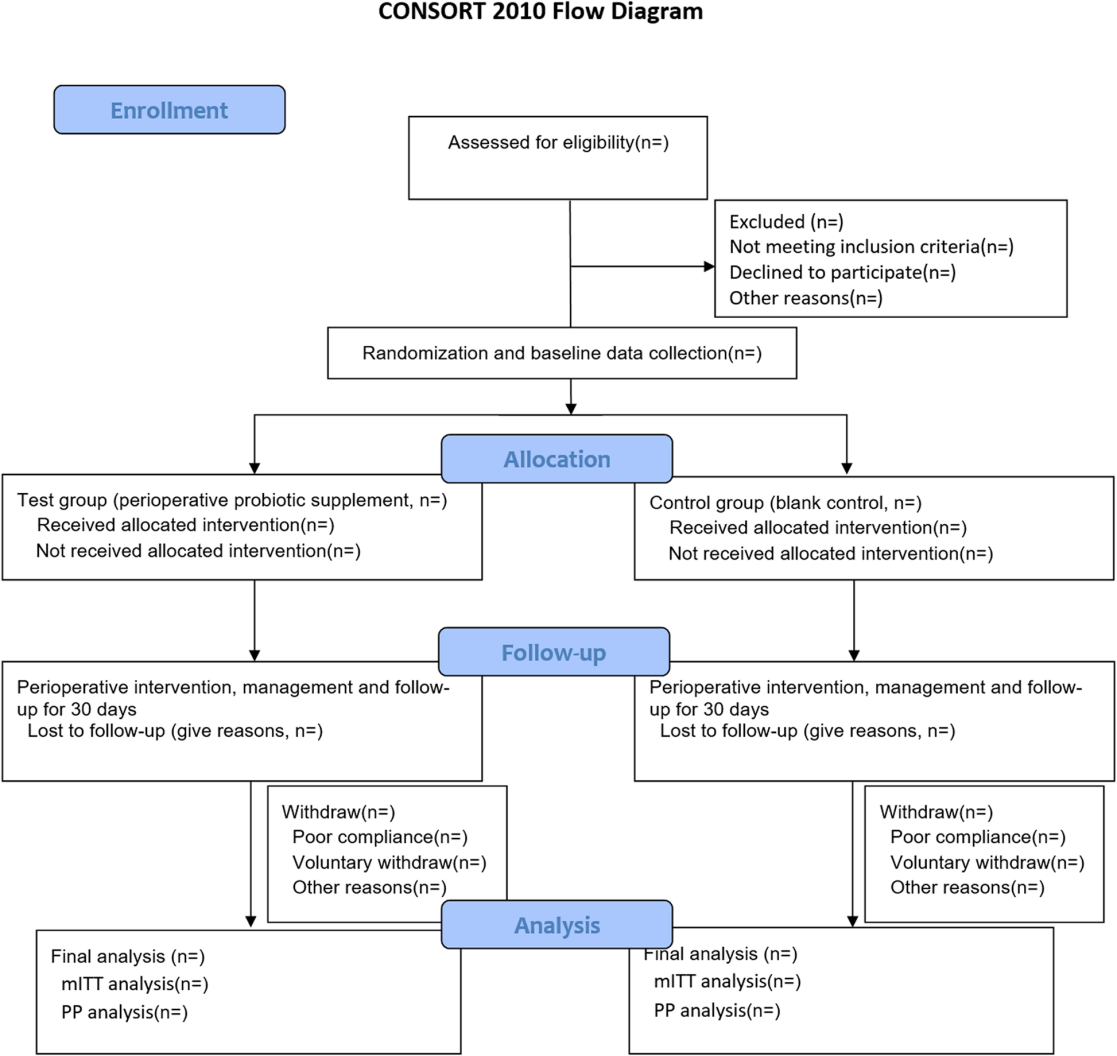
The CONSORT flowchart

The SPIRIT 2013 guidelines were followed for the construction of this report (Fig. 1) [22]. The CONSORT flowchart is shown in Fig. 2.

### Participants, interventions, and outcomes

#### Study setting

This study will enroll patients with AGC who have received NACT before laparoscopic or robotic radical gastrectomy. These patients will be enrolled from 22 hospitals, as listed in Table 1.

**Table 1:** The 22 participating surgical centers

Number	Centre	Department	Investigator
1*	The Affiliated Hospital of Qingdao University	Gastrointestinal Surgery	Yanbing Zhou
2	Qilu Hospital of Shandong University	Gastrointestinal Surgery	Wenbin Yu
3	Shandong Cancer Hospital	Gastrointestinal Surgery	Jie Chai
4	Shandong Provincial Hospital	Gastrointestinal Surgery	Leping Li
5	Yantai Yuhuangding Hospital	Gastrointestinal Surgery	Xixun Wang
6	Shandong Jining No.1 People's Hospital	Gastrointestinal Surgery	Xianqun Chu
7	Affiliated Hospital of Weifang Medical University	Gastrointestinal Surgery	Quanhong Duan
8	Weifang People's Hospital	Gastrointestinal Surgery	Jianjun Qu
9	Dongying People's Hospital	General Surgery	Hao Wang
10	Weihai Municipal Hospital	Gastrointestinal Surgery	Huanhu Zhang
11	Weihai Central Hospital	Gastrointestinal Surgery	Xinjian Wang
12	Rizhao People's Hospital	General Surgery	Xizeng Hui
13	Liaocheng People's Hospital	Gastrointestinal Surgery	Daogui Yang
14	Qingdao Municipal Hospital	Gastrointestinal Surgery	Shaofei Zhou
15	The Second Hospital of Shandong University	Gastrointestinal Surgery	Yinlu Ding
16	The People's Hospital of Jimo.Qingdao	General Surgery	Hongbo Wang
17	Binzhou People's Hospital	Gastrointestinal Surgery	Fengqiang Zhou
18	Binzhou Medical University Hospital	Gastrointestinal Surgery	Baoguang Hu
19	Jinan Central Hospital	Gastrointestinal Surgery	Peiming Guo
20	Yantai Yeda Hospital	General Surgery	Lixin Jiang
21	Shandong Provincial Qianfoshan Hospital	General Surgery	Guangyong Zhang
22	Rushan People’s Hospital	General Surgery	Qiang Pan

^*^ Sponsor Investigator

#### Eligibility criteria

Patients with AGC who have completed NACT will be screened for eligibility.

The inclusion criteria are as follows: 18–80 years old; both male and female; clinical stage of T3/4 N + evaluated by CT/MR/EUS at new diagnosis (before any anticancer treatment), completion of 2–4 cycles preoperative chemotherapy based on 5-FU (such as SOX, XELOX, FLOT, etc.) at 3–6 weeks before surgery; ASA grade of I-III; radical minimally invasive gastrectomy via a laparoscope or a DaVinci surgical system judged as possible; histologically confirmed gastric adenocarcinoma; ECOG score of 0 ~ 1; and provision of written informed consent before entering the study screening process.

The exclusion criteria are as follows: need for emergency surgery due to perforation and/or obstruction; receipt of antibiotics and/or glucocorticoids within 14 days before surgery; presence of bacterial infection and/or autoimmune disease and/or IBD; intolerance or allergy to probiotics; major upper abdominal surgery history (for example laparoscopic cholecystectomy); use of probiotics within 7 days before the intervention; current participation in other clinical trials; severe mental illness; inability to participate in this trial due to severe disease of other organs evaluated by researchers, such as severe cardiac insufficiency (LVEF < 30%, NYHA > II, severe arrhythmia, congestive heart failure, myocardial infarction within 6 months), liver dysfunction (Child‒Pugh C), renal dysfunction (need for hemodialysis); need for simultaneous surgery; lactation or pregnancy; refusal to participate in this trial.

Patients from the 22 hospitals will be evaluated, and those who meet the eligibility criteria will be invited to participate in the study. Patients will decide whether they are willing to participate in this study, and for those participants, written informed consent will be required.

The surgeons who perform the minimally invasive radical gastrectomy for the participants will also be evaluated. They should have performed more than 100 DaVinci robotic or laparoscopic radical gastrectomies and send an unedited operation radio to the sponsor investigators to verify their capacity.

### Interventions, surgery and perioperative management

Patients in the probiotic group will receive three probiotic capsules (BIFICO, live combined *Bifidobacterium*, *Lactobacillus* and *Enterococcus* capsules, Shanghai Sine Pharmaceutical Co. Ltd., Xinjinqiao Road, Shanghai, China) containing *Bifidobacterium longum, Lactobacillus acidophilus,* and *Enterococcus faecalis* twice per day. Each capsule will contain > 1 * 10^7^ colony-forming units (CFUs) of live bacteria. Patients in the control group will be managed with a blank control. The intervention duration will begin from the end of the last cycle of NACT to postoperative day (POD) 7 or the discharge day. Adherence to the intervention protocols will be checked by telephone before admission to the surgical departments and making the rounds of the wards after admission. If any adverse event associated with the intervention occurs, the intervention will be stopped after evaluation by the investigators.

All enrolled patients will adhere to a perioperative enhanced recovery after surgery (ERAS) protocol [23]. The ERAS program involves preoperative prehabilitation [24] (including physical and respiratory training, nutritional support and psychosocial treatment), no preoperative mechanical bowel preparation, fasting 6 h before surgery and oral glucose solution ingestion allowed until 2 h before surgery, intraoperative target-oriented liquid management, local anesthesia in the deep incision, general anesthesia combined with epidural anesthesia, early removal of the urinary catheter and abdominal drainage tube, early bedside activity, multimodal postoperative analgesia, and postoperative early food ingestion under the guidance of surgeons (from liquid to semi-liquid then solid food according to the tolerance of patients).

Laparoscopic or DaVinci robotic radical gastrectomy will be performed by surgeons from the 22 hospitals whose capacity evaluation is described above. Surgical procedures will be guided by *Japanese Gastric Cancer Treatment Guidelines 2021 (6 th edition)* [2]. The type of digestive tract reconstruction will depend on the habits, intraoperative status and past experience of the surgeons, and the choice of laparoscopy- or DaVinci-assisted surgery will depend on the patients’ choice.

Notably, all participants cannot intake any drugs containing probiotics and/or prebiotics during the study. Prophylactic antibiotic use is allowed during the operation and should be stopped within 24 h after surgery. Therapeutic antibiotics are allowed to be prescribed if postoperative infections occur, and the type and duration of antibiotic use are based on clinical experience and bacteriologic evidence.

### Outcomes

The primary outcome is the incidence of postoperative infectious complications, which are defined as bacterial infections occurring within 30 d after surgery. The definition of infectious complications is based on fever (≥ 38 °C), elevation of C-reactive protein (CRP), specific clinical symptoms of infection and positive bacterial culture. The diagnostic criteria for postoperative infections in this study are as follows. Pneumonia [25]: fever, cough, dyspnea, reduced arterial oxygen, typical pulmonary infiltrate on chest X-ray, positive culture from sputum or bronchoalveolar lavage. Urinary tract infection: obvious symptoms including frequent micturition, urgency to urinate, urodynia, leukocyturia, and a positive urine culture with > 10^5^ CFU/ml. Incision infection: redness or swelling of the incision, subcutaneous gas accumulation and purulent secretion and positive culture of incisional secretion [26]. Intraabdominal infection [27]: abdominal symptoms and positive signs such as abdominal pain, tenderness, rebound tenderness, radiographic evidence including intra-abdominal abscess, positive bacterial cultures from intra-abdominal smears or abdominal drainage fluid. Catheter-related blood stream infection: refer to the guidelines [28]. Systemic inflammatory response syndrome (SIRS): two or more of the following: a, temperature > 38 °C or < 36 °C; b, heart rate > 90/min; c, respiratory rate > 20/min or PaCO2 < 32 mm Hg (4.3 kPa); d, white blood cell count > 12 000/mm^3^ or < 4000/mm^3^ or > 10% immature bands [29]. The diagnosis of sepsis should follow the *Sepsis- 3 Consensus* [30].

Secondary outcomes for all participants include blood test results, such as the leukocyte count (WBC), percent of neutrophils (NEUT%), CRP and procalcitonin on PODs 1, 3, and 5; postoperative body temperature; length of postoperative hospital stay (LOS); total cost of hospitalization; 30-d readmission rate; 30-d mortality rate; duration of therapeutic antibiotic use; time to first flatus, first bowel movement and solid food tolerance; time to initiating adjuvant chemotherapy; EORTC QLQ-C30 score [31] on POD 30; PGSAS- 45 score [32] on PODs 3, 5 and 30; and visual analogue scale for pain evaluation on PODs 1, 3 and 5.

### Sample size

The sample size calculation was based on postoperative infection. Based on the results of our previous single-center RCT, the rates of postoperative infectious complications were 39.4% in the control group and 15.2% in the probiotic group. Given a significance one-sided level of *α* = 0.025 and test efficiency of 1 − *β* = 80%, a loss adjustment of 10% and a superiority threshold of 10%, the total sample requires at least 318 patients (159 in the test group and 159 in the control group on a 1:1 allocation ratio). The Test for Two Proportions (Non-Zero Null Hypothesis[Differences]) was used for sample size calculation (Hintze, J. (2011). PASS 11. NCSS, LLC. Kaysville, Utah, USA. www.ncss.com/).

### Recruitment

This study will enroll patients with AGC who received NACT before laparoscopic or robotic radical gastrectomy from 22 centers, as listed in Table 1. The launching conference was held to ensure that the details of the GISSG 2023–01 trial will be known by every investigator. There are three approaches to recruit patients: patients from outpatient departments, patients from oncology departments and patients referred from local hospitals who have finished NACT and plan to receive surgical treatment. All investigators from 22 centers were informed that the clinical trial had begun and that the participants should know all details about the trial to obtain an adequate number of participants. The enrollment of the first patient was started in April 2023, and it is anticipated that the deadline for the recruitment of the patients will be in April 2024. Ultimately, 318 patients who meet the inclusion criteria will be included in this trial and randomized into a probiotic group and a control group in a 1:1 ratio. A conference for all investigators will be held bimonthly for communication about difficulties and experiences during recruitment and for deciding whether to improve the study protocol.

### Assignment of interventions

A central dynamic, stratified strategy was adopted for the aim of randomization. The sequence of randomization was generated by a certain statistician who was independent of this trial using the method of Pocock-Simon minimization by SAS 9.3 (SAS Institute Inc., Cary, NC, USA) and stratified by age (< 65 years old or ≥ 65), tumor location (upper, middle or lower) and preoperative chemotherapy cycles (2 or ≥ 2 cycle). The stratification information provided by the investigators was submitted to the statistician who was responsible for executing randomization. Consequently, the allocation information will then be sent back to each participating site, and investigators will assign given interventions. The statistician is the only one who will know the allocation sequence to ensure that the sequence is concealed until the interventions are assigned.

Due to the open-label design of this study, no one will be blinded after assignment to interventions. After receiving the allocation information, investigators from every participating center will assign the given interventions to participants.

## Data collection, management, and analysis

### Data collection methods and management

All data will be entered into an electronic data capture (EDC) system and recorded on a paper case report form (CRF). Subaccounts will be assigned to participating by the sponsor-investigator, and every modification will be recorded in the background of the EDC to ensure facticity. The CRF should be sent back to sponsor-investigator for cross check. An independent statistician is responsible for collecting the data and is unaware of the participants'group assignments.

### Statistical methods

The Shapiro‒Wilk test will be used to verify the normality of the quantitative variables. To describe the quantitative variables, the mean and SD will be used, and the median and interquartile range will be used for those variables that do not follow a normal distribution. For qualitative variable descriptions, frequencies and percentages will be used. A contrast test of proportions will be based on the *χ*^*2*^ test or Fisher’s exact test. Quantitative variables will be analyzed by Student’s *t* test or two-way repeated-measures analysis. The Mann‒Whitney *U* test will be used for variables that do not follow a normal distribution. The results will be analyzed using SPSS version 24 (IBM, Armonk, NY, USA), and *P* < 0.05 will be considered statistically significant. The mean of each variable will be used to replace missing data.

A modified intention-to-treat analysis (mITT) and per-protocol (PP) analysis will be used to compare outcomes, but the conclusion will be drawn from mITT analysis.

The mITT population is defined as subjects who are screened by investigators according to the inclusion and exclusion criteria, randomized into certain groups and receive at least one intervention. The PP population is defined as those who complete the given intervention, adhere to the study protocol and complete the data collection. Subgroup analysis will be executed based on the operation platform (laparoscope or DaVinci surgical robot), preoperative chemotherapy regimens (SOX, DOS, XELOX, FLOT or others), GI restruction and resection range (proximal, distal or total gastrectomy).

## Monitoring

### Data monitoring and auditing

A data monitoring committee (DMC) consisting of surgeons, statisticians and ethics experts was established to improve the quality of this trial. The DMC remains independent of the research team of the project in order to objectively evaluate the effectiveness and safety. The interim analyses will be performed when half of the estimated participants (n = 159) are enrolled, and then important decisions will be made regarding whether to “continue the trial”, “continue the trial after adjusting the protocol” or “terminate the trial”. The results of the interim analysis will be released to all investigators. The auditing will be executed monthly by DMC based on CRFs and the EDC system.

### Harms

A few studies have reported that probiotics may cause infections and other adverse clinical events in critically ill patients [33, 34]. *Enterococcus faecalis*, which is used in the intervention, is a special probiotic that has also been reported as an opportunistic pathogen. However, the virulence factor detection of the *Enterococcus faecalis* species used in this study proved its safety with no virulence factor gene *IS16*, hyaluronidase gene, asa1, asa37 and hemolysin A. Our previous single-center study showed the safety of the probiotic capsule. If any adverse effects related to the intervention occur, they (as well as the solutions) will be recorded in detail in the CRF and EDC. If the intervention shows obvious harm to participants, the DMC has a full right to stop this study after informing all investigators.

### Ethics and dissemination

After reviewing the study protocol, informed consent, declaration of interest conflict and other files, the GISSG 2023–01 study was approved by the Ethics Coordinating Committee of the Affiliated Hospital of Qingdao University for implementation (QYFYEC2023 - 14), which is also responsible for supervision of this study. The implementation of this study will be in accordance with the *Declaration of Helsinki 2013*. Investigators should obtain informed consent from participants after explaining the study objectives, methodology, and possible benefits and harms. Any information that can identify participants, such as name and ID number, must be kept confidential to protect patients’ privacy unless under special circumstances as required by law. All data will be acquired only by study investigators who have signed a confidential disclosure agreement. The data will be used for analysis and finally concluded in the form of published articles only with authorization by the sponsor-investigator. Unless the periodical office places a request for raw data disclosure, the data will not be displayed to the public.

### Strengths and limitations

This is the first multicenter, large-sample RCT to comprehensively evaluate the effects of perioperative probiotic supplements on the short-term clinical outcomes of patients undergoing laparoscopic or robotic radical gastrectomy after neoadjuvant chemotherapy. We plan to observe postoperative infections, blood inflammation parameters, recovery of gastrointestinal function and quality of life to investigate the overall effects of probiotics on gastric cancer patients receiving minimally invasive gastrectomy after NACT. NACT may cause susceptibility to bacterial infection, nutrition risk and other clinical features, which generate some difficulties and particularities for perioperative management. We hope to summarize some experiences from this study targeting the intestinal microbiota to improve perioperative care for this special group of patients. A limitation is that the subjective consciousness of the surgeon may lead to deviations in the results because this is an open-label trial.

## Discussion

NACT has been widely used in treating AGC with advantages in improving survival, shrinking tumors before surgery and increasing the radical resection rate [5, 6]. However, Schuhmacher et al. reported that patients with GC who received NACT developed more postoperative complications than those who received surgery first (27.1% *versus* 16.2%) [7]. Our previous study showed that NACT may impair gut barrier function by downregulating the expression of tight junction-related proteins (Claudin- 1, ZO- 1, and Occludin), shortening intestinal villi and disturbing the intestinal microbiota balance [8]. Probiotics and their metabolites can not only inhibit the adhesion of pathogenic bacteria by promoting the secretion of antimicrobial peptides from intestinal epithelial cells but also strengthen the tight junctions of the mucosal epithelium [35, 36]. Correia et al. systematically reviewed the main functions of probiotics in GI surgery, including regulating intestinal flora and increasing the abundance of the ingested probiotics; preventing pathogenic bacterial adhesion through competitive inhibition, decreasing epithelial permeability and improving the gene expression of mucosal repair; inhibiting intestinal inflammation and increasing the activity of natural killer cells and inducing the secretion of anti-inflammatory cytokines [37]. Therefore, the introduction of probiotics into the perioperative management of AGC patients receiving NACT may reverse the adverse effects of NACT and surgical stress.

Yuan et al. found that *Bifidobacterium infantis* can increase the expression of PCNA, reduce the expression of NF-κB and proinflammatory factors and decrease the myeloperoxidase (MPO) concentration in 5-FU-induced intestinal mucositis in rats [9]. Justino et al. demonstrated that *Lactobacillus acidophilus* can maintain the normal histologic structure of the intestinal mucosa, increase GSH concentration, decrease MPO activity and nitrite concentrations, inhibit the secretion of cytokines (TNF-α, IL- 1β, and CXCL- 1) and accelerate gastric emptying and gastrointestinal transit [10]. Zheng et al. investigated the effects of probiotics on short-term outcomes in patients undergoing radical gastrectomy and a rat model with gastrectomy. They found that probiotic supplementation could significantly relieve postoperative inflammation, enhance immunity, resume gut microbiota balance and promote postoperative recovery. Similar results were also found in a rat model. Furthermore, probiotic compound administration could downregulate the inflammatory and permeability signaling pathways in the intestinal tissue in rats [38]. Our previous single-center RCT found that AGC patients undergoing minimally invasive gastrectomy after NACT might gain benefits from appropriate supplementation with perioperative probiotics, which may be related to fewer postoperative infections, faster recovery of gastrointestinal function and lower laboratory parameters of systemic inflammation [21].

Overall, perioperative probiotic supplementation is safe and can improve short-term outcomes after major abdominal surgery by reducing postoperative infection and enhancing postoperative recovery. It is expected that the results of this trial can provide high-level evidence-based medical support and have clinical value for perioperative probiotic supplementation in AGC patients undergoing minimally invasive radical gastrectomy after NACT.

### Trial status

Patient recruitment was completed now. The trial was in the stage of data collection at each participating site when we firstly submitted this manuscript and was ended during peer review. To ensure the authenticity, scientificity and safety of the participants, investigator conferences and DMC supervision were conducted on schedule. The study protocol was modified to version 1.0.

## Supplementary Information

Supplementary Material 1.

## Data Availability

Data from this randomized controlled study were unavailable at the time of publication. Individual participant data are available upon request.

## Abbreviations

RCT: Randomized controlled trial
CRF: Case report form
EDC: Electronic data capture
AGC: Advanced gastric cancer
NACT: Neoadjuvant chemotherapy
DMC: Data monitoring committee
WBC: Leukocyte count; NEUT%: percentage of neutrophils
CRP: C-reactive protein
PCT: Procalcitonin
POD: Postoperative day
